# Supplementary material for: New Insight into the Genotype-Phenotype Correlation of PRPH2-Related Diseases Based on a Large Chinese Cohort and Literature Review
Source: Int J Mol Sci. 2023 Apr 4;24(7):6728. doi: 10.3390/ijms24076728 (PMC10095211; doi:10.3390/ijms24076728)
Supplement: Supplementary file 1 [file ijms-24-06728-s001.zip › WangYW-PRPH2-Sup Figure S1 sub.pdf]

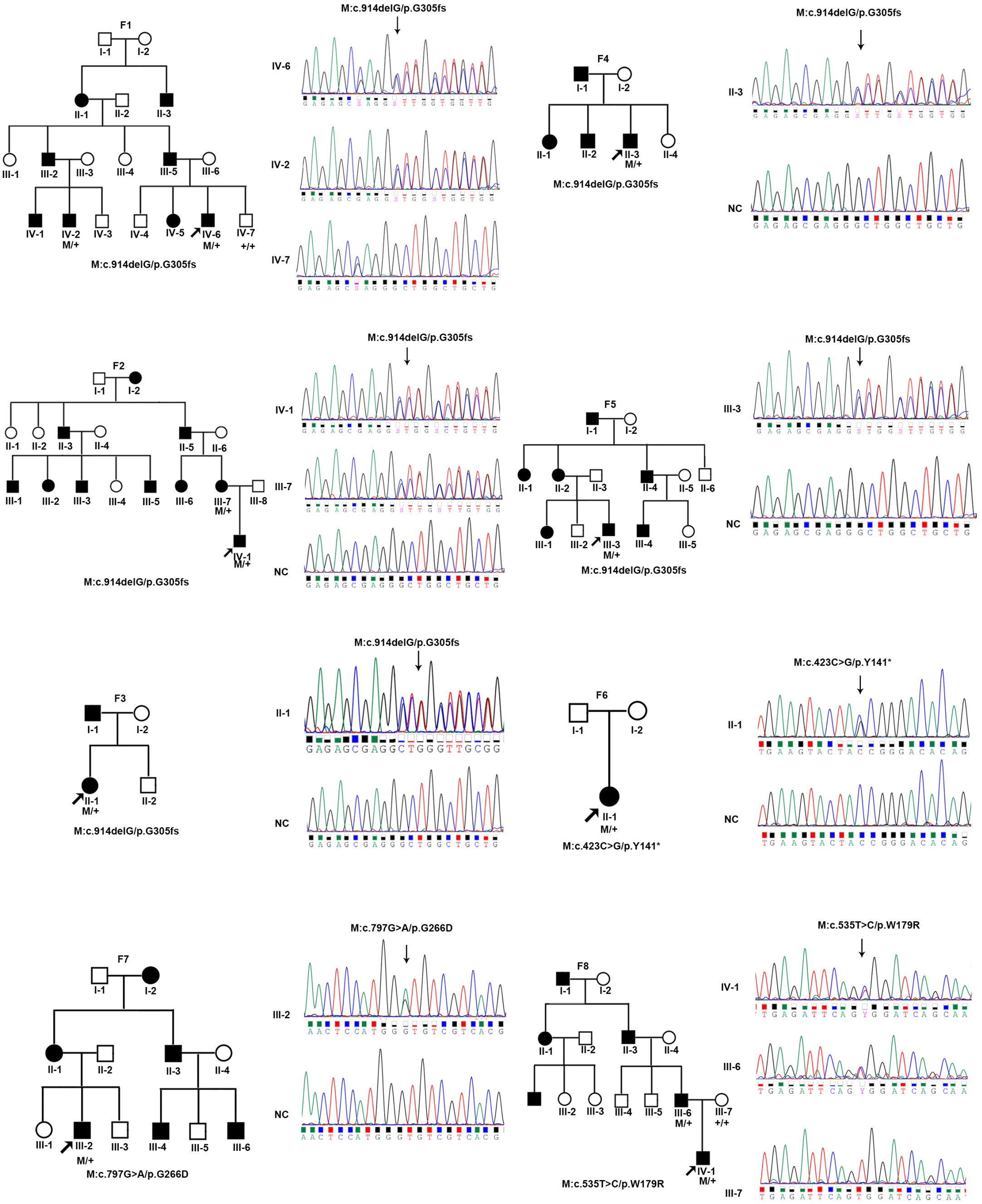
F1
I-1
I-2
II-1
II-2
II-3
III-1
III-2
III-3
III-4
III-5
III-6
IV-1
IV-2
M/+
IV-3
IV-4
IV-5
IV-6
M/+
IV-7
+/+
M:c.914delG/p.G305fs
M:c.914delG/p.G305fs
IV-6
IV-2
IV-7
F4
I-1
I-2
II-1
II-2
II-3
M/+
II-4
M:c.914delG/p.G305fs
M:c.914delG/p.G305fs
II-3
NC
F2
I-1
I-2
II-1
II-2
II-3
II-4
II-5
II-6
III-1
III-2
III-3
III-4
III-5
III-6
III-7
M/+
III-8
IV-1
M/+
M:c.914delG/p.G305fs
M:c.914delG/p.G305fs
IV-1
III-7
NC
F5
I-1
I-2
II-1
II-2
II-3
II-4
II-5
II-6
III-1
III-2
III-3
M/+
III-4
III-5
M:c.914delG/p.G305fs
M:c.914delG/p.G305fs
III-3
NC
F3
I-1
I-2
II-1
M/+
II-2
M:c.914delG/p.G305fs
M:c.914delG/p.G305fs
II-1
NC
F6
I-1
I-2
II-1
M/+
M:c.423C>G/p.Y141*
M:c.423C>G/p.Y141*
II-1
NC
F7
I-1
I-2
II-1
II-2
II-3
II-4
III-1
III-2
M/+
III-3
III-4
III-5
III-6
M:c.797G>A/p.G266D
M:c.797G>A/p.G266D
III-2
NC
F8
I-1
I-2
II-1
II-2
II-3
II-4
III-2
III-3
III-4
III-5
III-6
M/+
III-7
+/+
IV-1
M/+
M:c.535T>C/p.W179R
M:c.535T>C/p.W179R
IV-1
III-6
III-7

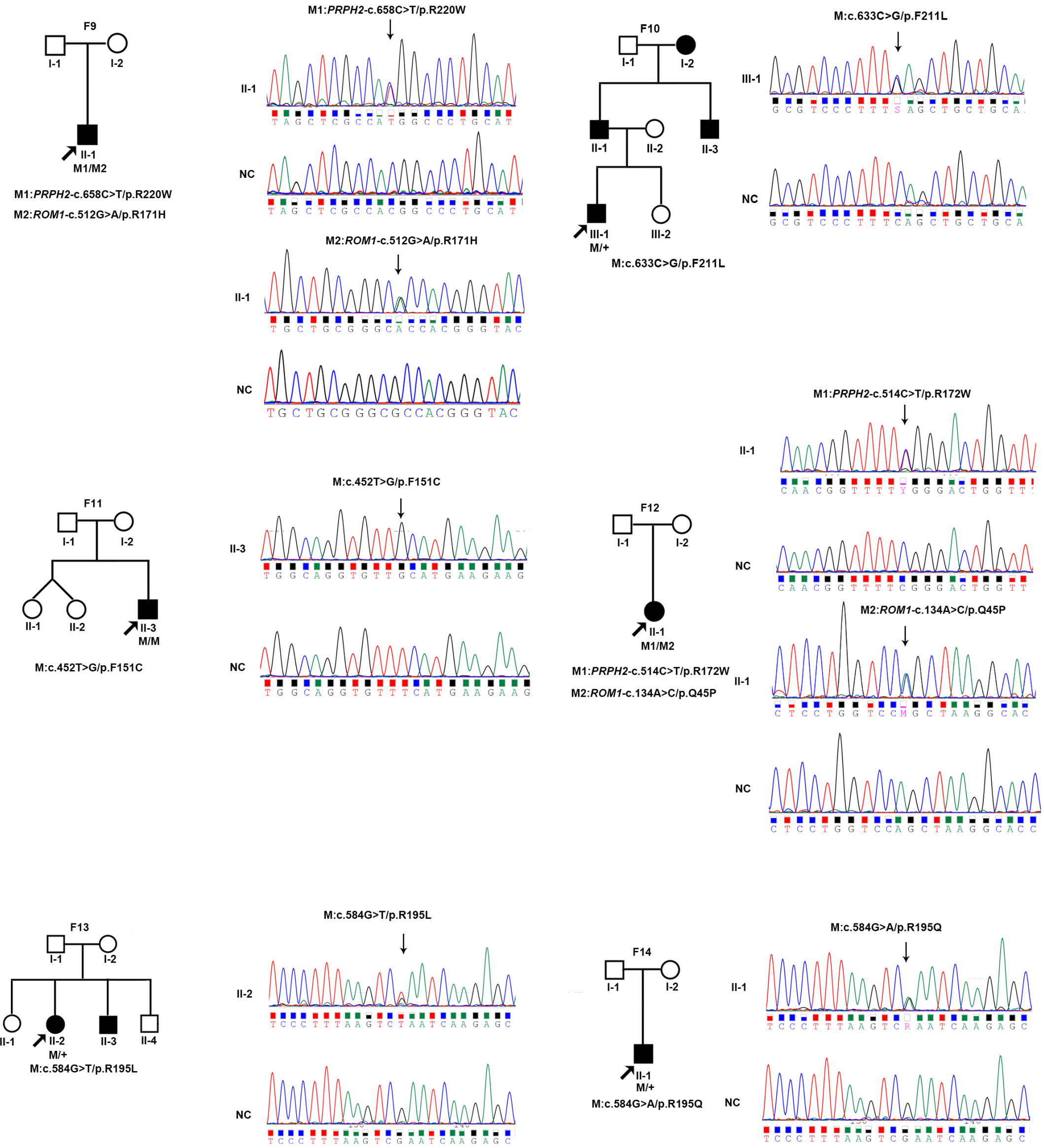

Supplementary Figure S1. Sanger sequences and the corresponding pedigrees of 14 with pathogenic or likely pathogenic variants in *PRPH2* observed in this study.
