# Supplementary material for: New Insight into the Genotype-Phenotype Correlation of PRPH2-Related Diseases Based on a Large Chinese Cohort and Literature Review
Source: Int J Mol Sci. 2023 Apr 4;24(7):6728. doi: 10.3390/ijms24076728 (PMC10095211; doi:10.3390/ijms24076728)
Supplement: Supplementary file 1 [file ijms-24-06728-s001.zip › WangYW-PRPH2-Sup Figure S2 sub.pdf]

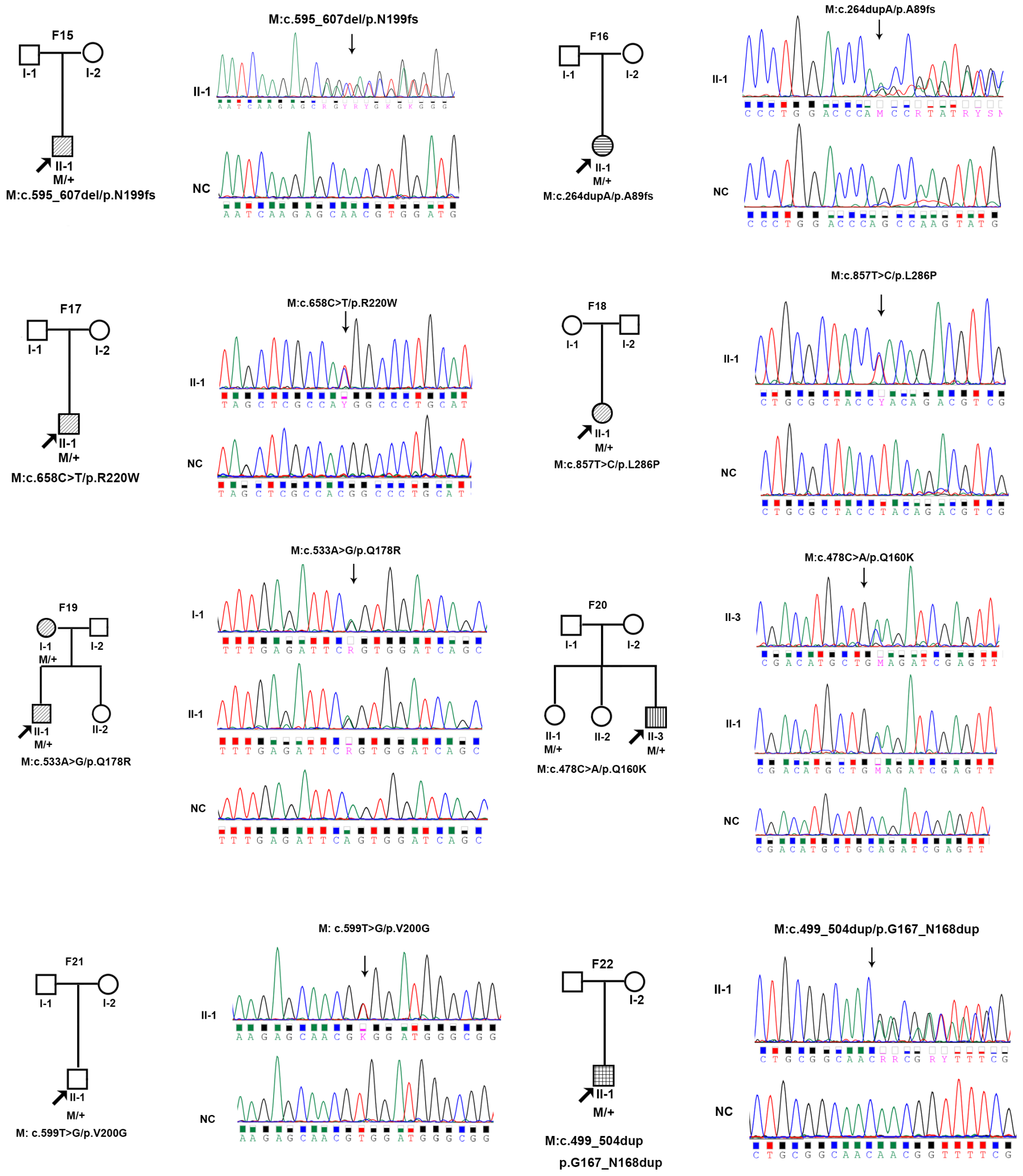

Supplementary Figure S2. Sanger sequences and the corresponding pedigrees of eight with variants uncertain significance in *PRPH2* observed in this study.
