# Supplementary material for: New Insight into the Genotype-Phenotype Correlation of PRPH2-Related Diseases Based on a Large Chinese Cohort and Literature Review
Source: Int J Mol Sci. 2023 Apr 4;24(7):6728. doi: 10.3390/ijms24076728 (PMC10095211; doi:10.3390/ijms24076728)
Supplement: Supplementary file 1 [file ijms-24-06728-s001.zip › WangYW-PRPH2-Sup Figure S3 sub.pdf]

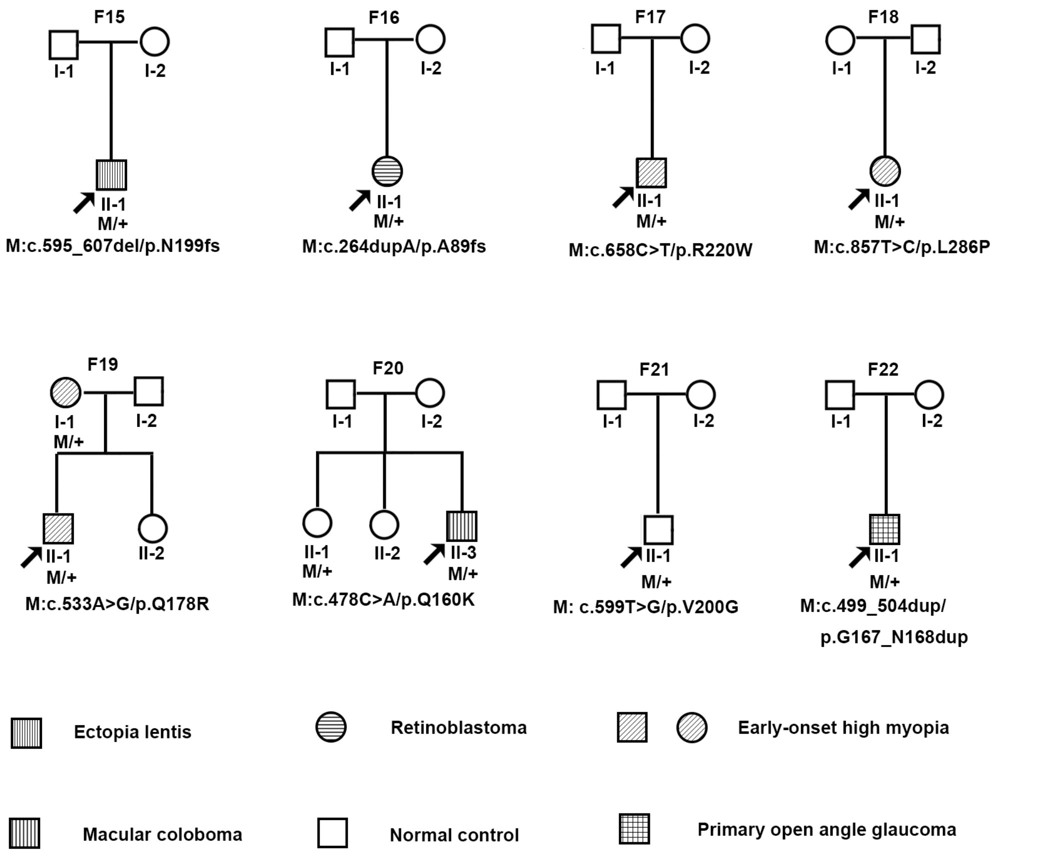

Supplementary Figure S3. The pedigrees of eight families identified with *PRPH2* variants uncertain significance and presented unrelated phenotypes in this study. Squares represent male individuals and circles indicates female. Affected patients were showed by squares or circles with different stripe shapes and the proband of each family was indicated by arrows. Family numbers puts on the top of the pedigrees while variants are listed under the pedigrees.
