# Supplementary material for: New Insight into the Genotype-Phenotype Correlation of PRPH2-Related Diseases Based on a Large Chinese Cohort and Literature Review
Source: Int J Mol Sci. 2023 Apr 4;24(7):6728. doi: 10.3390/ijms24076728 (PMC10095211; doi:10.3390/ijms24076728)
Supplement: Supplementary file 1 [file ijms-24-06728-s001.zip › WangYW-PRPH2-Sup Figure S4 sub.pdf]

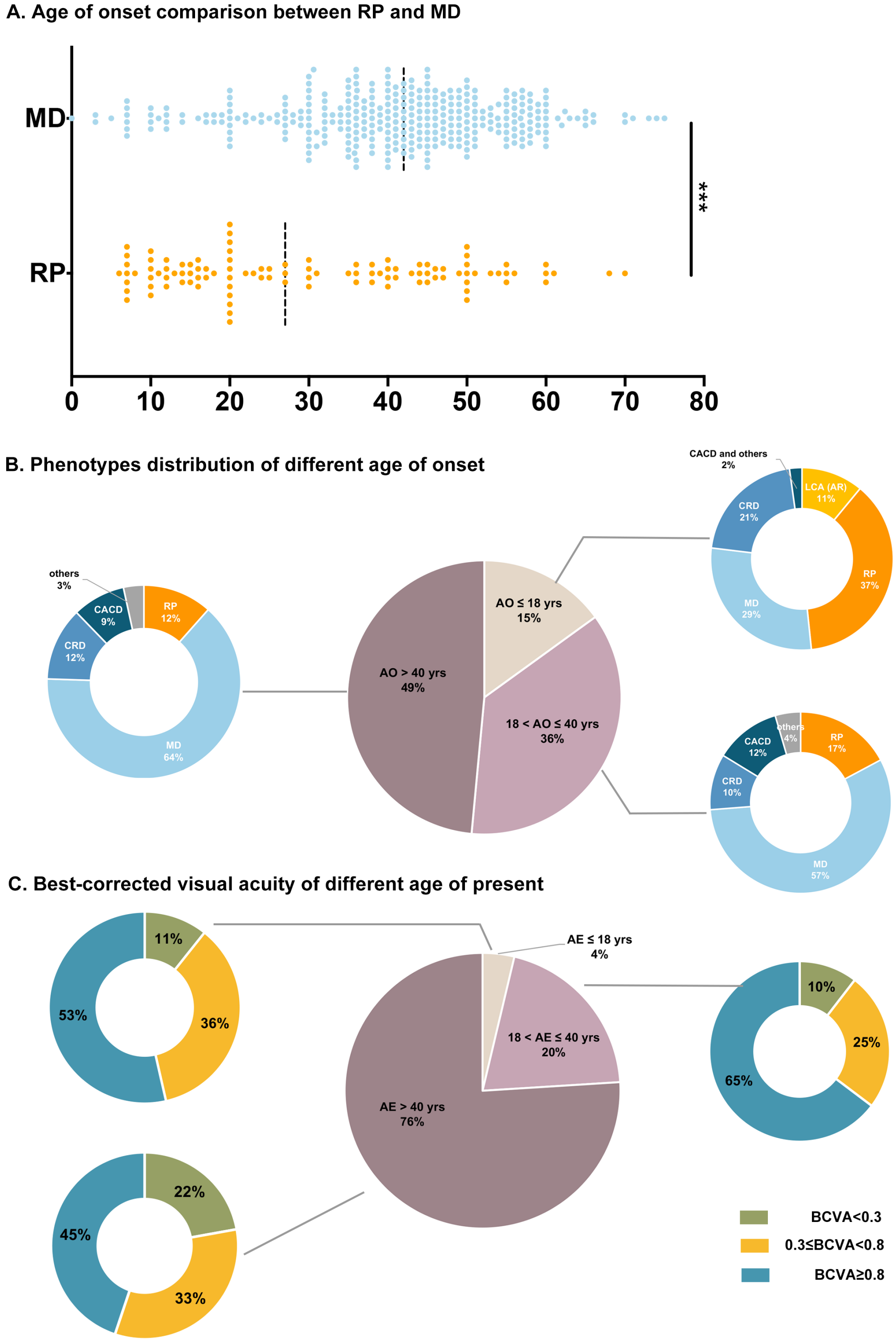

Supplementary Figure S4. The clinical characteristics of *PRPH2*-associated retinopathy summarized based on comprehensive literature review. (A). A total of four hundred and forty-five patients in previous study had available age of onset, in which age of onset of patients with RP was significantly earlier than those with MD (*** represented P<0.0001). (B) Divided patients in literature with available age of onset into three groups, it was found that the proportion of Leber congenital

amaurosis and retinitis pigmentosa was more among the patients with age of onset no more than 18 years old than those develop PRPH2-associated retinopathy latter. (C) More than half of patients in previous study were conducted with ocular examinations after 40 years old. the adult patients who were no older than 40 when examined had BCVA no older than 0.3, which were less than those older than 40 years old had BCVA no older than 0.3) with significantly statistic differences (*P*=0.001).
